# Determination of Amide *cis*/*trans* Isomers in *N*‐Acetyl‐d‐glucosamine: Tailored NMR Analysis of the *N*‐Acetyl Group Conformation

**DOI:** 10.1002/cbic.202200338

**Published:** 2022-07-11

**Authors:** Yan Xue, Gustav Nestor

**Affiliations:** ^1^ Department of Molecular Sciences Swedish University of Agricultural Sciences Uppsala BioCenter, P.O. Box 7015 750 07 Uppsala Sweden

**Keywords:** conformational analysis, isotopic labelling, *N*-acetylglucosamine, NMR spectroscopy

## Abstract

*N*‐Acetyl‐d‐glucosamine (GlcNAc) is one of the most common amino sugars in nature, but the conformation of its *N*‐acetyl group has drawn little attention. We report herein the first identification of NH protons of the amide *cis* forms of *α*‐ and *β*‐GlcNAc by NMR spectroscopy. Relative quantification and thermodynamic analysis of both *cis* and *trans* forms was carried out in aqueous solution. The NH protons were further utilized by adapting protein NMR experiments to measure eight *J*‐couplings within the *N*‐acetyl group, of which six are sensitive to the H2‐NH conformation and two are sensitive to the amide conformation. For amide *cis* and *trans* forms, the orientation between H2 and NH was determined as *anti* conformation, while a small percentage of *syn* conformation was predicted for the amide *trans* form of *β*‐GlcNAc. This approach holds great promise for the detailed conformational analysis of GlcNAc in larger biomolecules, such as glycoproteins and polysaccharides.

## Graphical abstract

**The** ***N*** **‐acetyl group** of *N*‐acetyl‐d‐glucosamine (GlcNAc) is defined by two torsion angles, *θ_1_* and *θ_2_*, where *θ_2_* defines the amide bond orientation which enables *cis*‐*trans* isomerization (CTI). This is the first identification of NH protons of the amide *cis* isomers of *α*‐ and *β*‐GlcNAc, which has allowed conformational analysis of these isomers based on *J*‐couplings within the *N*‐acetyl group.

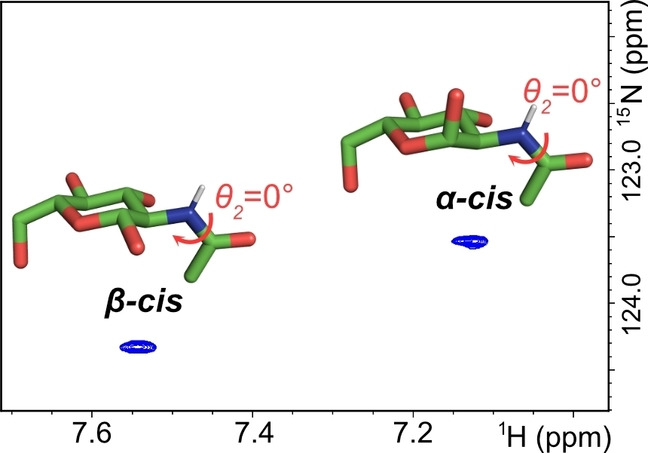

## Introduction

Amino sugars are widely distributed in nature and function as key components of glycoproteins, glycolipids, and glycosaminoglycans.^[1]^ *N*‐Acetyl‐d‐glucosamine (GlcNAc), one of the most common amino sugars in nature, appears as building blocks in many polysaccharides and glycoconjugates such as chitin, hyaluronic acid (HA), and peptidoglycan, which are broadly involved as biological and structural components of cell walls and extracellular matrices.[^2^, ^3^] GlcNAc is an essential constituent in both *O*‐ and *N*‐glycosylation and it is involved in accommodating various biosynthesis and signaling pathways in diverse organisms including animals, bacteria, and fungi.[^4^, ^5^, ^6^, ^7^] Moreover, GlcNAc is tightly associated with a large number of human diseases, for example as a modulator of intracellular signaling, where GlcNAc regulates the insulin pathway in adipocytes.^[2]^

Since the conformations of GlcNAc polysaccharides mainly depend on glycosidic‐linkage geometry and pyranosyl ring conformation,^[8]^ structural changes and kinetics of the *N*‐acetyl group of GlcNAc and its amide linkage have drawn little attention.^[9]^ However, the *N*‐acetyl group can adopt different conformations that will determine its participation in both intra‐ and intermolecular hydrogen bonds and water bridges, which might also be critical for the general geometry of polysaccharides. Amide *cis*‐*trans* conformation is a key determinant for amide linkages and *cis*‐*trans* isomerization (CTI) is considered a crucial biological exchange process, especially in peptide linkages.^[10]^ The ability of the amide nitrogen atom to delocalize its electron lone pair and the consequent partial double bond character between nitrogen and carbonyl carbon hinder free rotation around the C−N bond, resulting in *cis* and *trans* isomers, where *trans* is energetically favored over *cis*.^[11]^ However, it has been shown that a certain amount of amide *cis* conformation exists in small organic compounds as well as in larger biomolecules.[^12^, ^13^, ^14^]

**Scheme 1:**
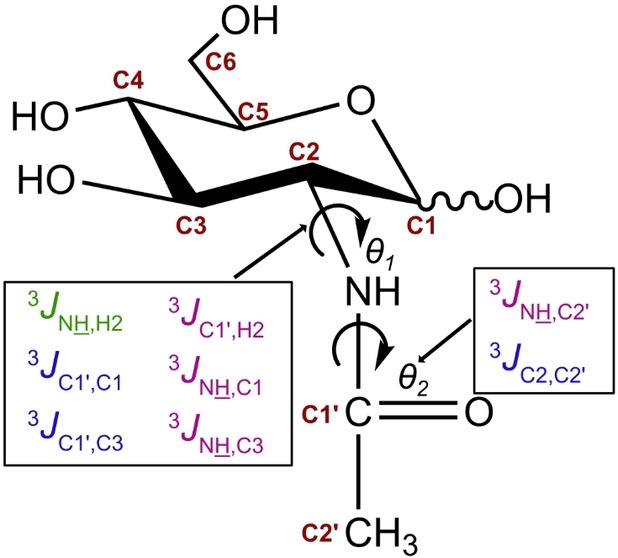
*J*‐couplings related to the torsion angles *θ*_1_ and *θ*_2_ in *α*‐ and *β*‐GlcNAc.

**Scheme 2:**
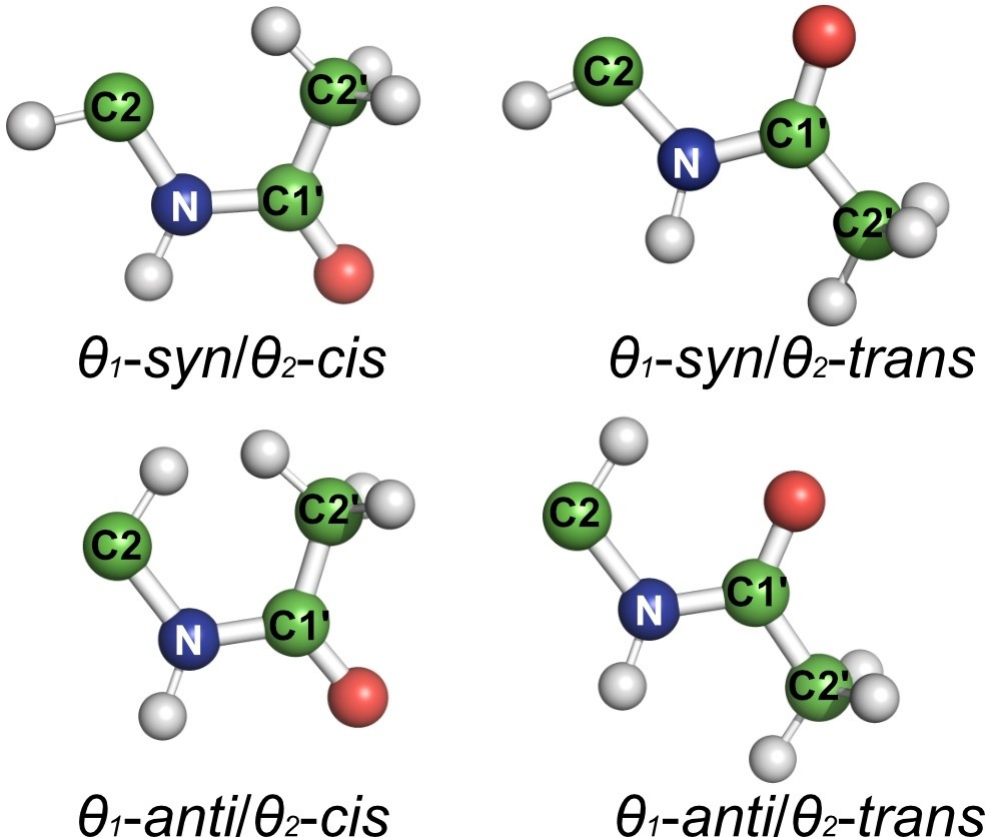
Four conformations that are related to the torsion angles *θ*_1_ and *θ_2_* in the *N*‐acetyl side chain.

In GlcNAc, the two torsion angles *θ_1_* (H2−C2−N−H) and *θ_2_* (C2−N−C1′−C2′), as shown in Scheme 1, define the conformation of the *N*‐acetyl group. The amide bond defined by *θ_2_* enables CTI and the activation energy for *cis*‐*trans* interconversion has been determined to about 20 kcal/mol in GlcNAc methyl glycosides,^[9]^ which makes the exchange slow enough to observe separate NMR signals for the *cis* and *trans* forms at room temperature. Prior studies show that the amide bond in GlcNAc is predominantly in the *trans* conformation (the relative orientation of C2 and C2′, see Scheme 2), with only about 1.8 % in the *cis* conformation at 42 °C.^[9]^ However, surveys on glycoprotein X‐ray crystal structures found that as much as 6–12 % of the GlcNAc residues populate the *cis* conformation,[^9^, ^15^] although a substantial amount of these structures are severely twisted or may be due to an erroneous interchange of the carbonyl oxygen and the methyl carbon, which indicates an overestimation of the amount of *cis* conformations.[^15^, ^16^] Thus the amide *cis* conformation may play an important role in certain glycoconjugates, although characterization of the *cis* form in monomeric GlcNAc remains challenging due to the low abundance in aqueous solution.

The C2‐N bond, on the other hand, is more flexible than the amide bond. The *anti* conformation (defined by the relative orientation of H2 and NH, see Scheme 2) is considered to be the preferred one in GlcNAc monomers, as predicted by molecular dynamic (MD) simulations and NMR data.[^9^, ^17^] However, deviations from the *anti* conformation can be crucial for the possibility of NH hydrogen bond interactions. For example, GlcNAc in HA oligosaccharides is known to be in a H2‐NH *anti* conformation,^[18]^ but it is still unclear to what extent the polymer deviates from the *anti* conformation to form a hydrogen bond between the amide proton and a neighboring carboxylate group,^[19]^ or even contain a significant fraction of *syn* conformation.^[20]^ The activation energy for *anti*‐*syn* interconversion of GlcNAc was determined by density functional theory (DFT) calculations to 5–10 kcal/mol,^[9]^ and with small chemical shift differences between the two forms, no separate NMR signals of the two forms can be observed.

Spin‐spin coupling constants (also known as *J*‐couplings), together with NOEs, are the most important NMR tools for 3D structure determination of biomolecules. Previous work on GlcNAc has focused on the homonuclear H2‐NH coupling constant (^3^ *J*_N_ ${}_{\underline{H}}$_,H2_) to distinguish between *anti* and *syn* conformation.[^17^, ^21^, ^22^, ^23^] More recently, Hu et al. showed the advantage of using a set of *J*‐couplings to determine the conformation of both torsion angles (*θ_1_* and *θ_2_*).^[24]^ The same group also showed the presence of the amide *cis* conformation from ^13^C NMR spectra of GlcNAc methyl glycosides with site‐specific ^13^C‐labeling and characterized the *cis*‐*trans* equilibrium and the exchange kinetics.^[9]^

In this study, the amide protons of the GlcNAc *cis* forms were observed for the first time and amide protons of both *cis* and *trans* forms were investigated by NMR spectroscopy. A series of NMR experiments were utilized to measure *J*‐couplings within the *N*‐acetyl group of uniformly ^13^C,^15^N‐labeled GlcNAc. ^13^C,^15^N‐labeling enabled the measurement of eight vicinal scalar coupling constants (one ^3^ *J*_HH_, three ^3^ *J*_CC_, and four ^3^ *J*_CH_) within the *N*‐acetyl group, of which six are sensitive to the *θ_1_* angle and two are sensitive to the *θ_2_* angle (Scheme 1). The *J*‐couplings were used to identify the preferred *N*‐acetyl conformation of both *cis* and *trans* forms of GlcNAc.

## Results and Discussion

### Amide protons of minor *cis* conformers

**Figure 1:**
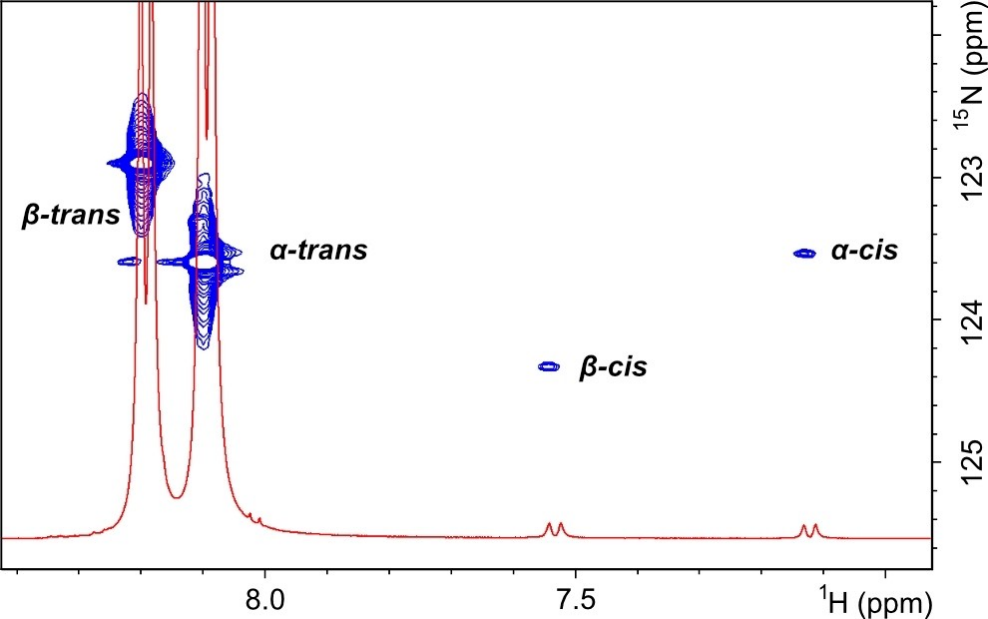
Selected region of 2D‐[^1^H, ^15^N] HSQC and 1D‐^1^H spectra of GlcNAc at 25 °C. Amide protons of *α*‐ and *β*‐GlcNAc in *cis* and *trans* forms are highlighted.

**Figure 2:**
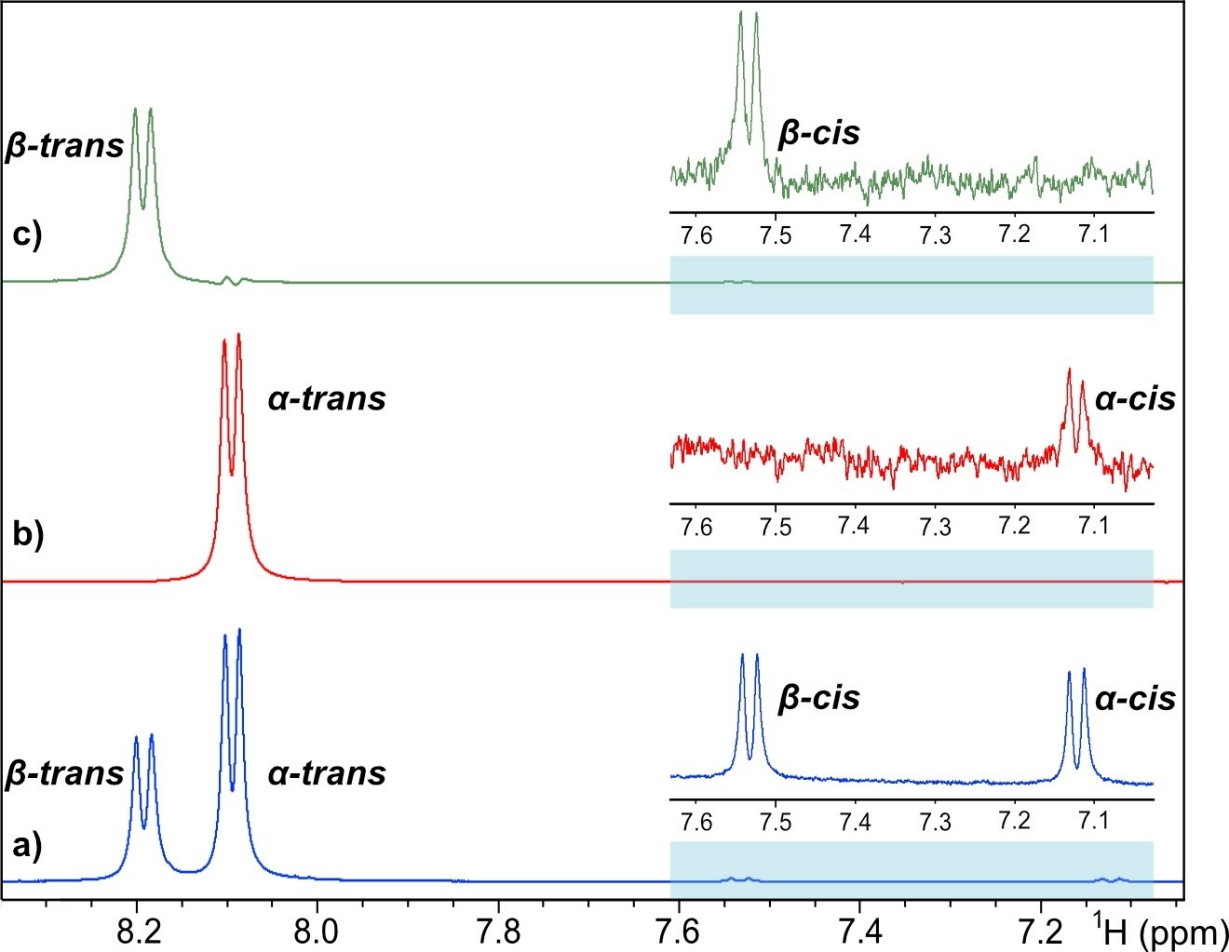
Selected region of a) 1D‐^1^H spectrum with excitation sculpting; b) 1D selective EXSY spectrum targeted at the *α*‐*trans* signal; c) 1D selective EXSY spectrum targeted at the *β*‐*trans* signal.

The *trans* conformers of the *α*‐ and *β*‐anomer of GlcNAc, which constitute >98 % at room temperature, could readily be identified from amide proton cross‐peaks in the ^1^H,^15^N‐HSQC spectrum (Figure 1) of uniformly ^13^C,^15^N‐labeled GlcNAc in 90 % H_2_O/10 % D_2_O. This pattern is similar to previous studies on GlcNAc using ^1^H,^15^N‐HSQC.[^25^, ^26^] However, closer examination of the GlcNAc ^1^H,^15^N‐HSQC revealed two weak cross‐peaks, which correlated with doublets at 7.12 and 7.53 ppm in the ^1^H NMR spectrum (Figure 1). These species constituted about 0.5 % each, compared to the sum of the *trans* forms. By performing 1D selective EXSY experiments, exchange correlations with the *trans* forms could be observed (Figure 2), so that the doublet at 7.12 ppm correlated with the *α‐trans* form and the doublet at 7.53 ppm correlated with the *β‐trans* form. Comparison of the ^13^C chemical shifts of the *cis* forms (*vide infra*) with the earlier observation of the *cis* forms^[9]^ showed identical results and we could thus assign the two weak ^1^H‐^15^N cross‐peaks to the *α‐cis* (7.12 ppm) and *β‐cis* (7.53 ppm) forms of GlcNAc.

### Chemical shifts

In order to distinguish signals arising from *cis* forms with very low abundance, several NMR experiments were conducted to assign the chemical shifts of the different GlcNAc forms. The assignments of ^13^C resonances were obtained from ^1^H,^13^C‐CT‐HSQC, (H)C(C)H‐TOCSY and HNCACB experiments, whereas the assignments of ^1^H resonances were also obtained from ^1^H,^15^N‐HSQC‐TOCSY and ^1^H‐selective experiments (Figure S1).

The ^1^H chemical shifts are affected by the conformation of the *N*‐acetyl side chain and the *α/β* anomeric configuration as shown in Tables 1 and 2. Amide protons were most affected and the amide *cis* forms shift upfield (up to 1.0 ppm) compared to the *trans* form with the same anomeric configuration. Meanwhile, amide protons of the *β* anomer have higher chemical shifts compared to *α* anomer with the same amide conformation. Similarly, the H2 signals of the *cis* forms are shifted upfield (0.3–0.4 ppm) compared to the *trans* forms. However, H2 resonances of both *trans* and *cis* forms experienced more downfield chemical shifts in the *α* anomer than in the *β* anomer, which is the opposite of the amide protons. The H2 and NH protons are close to the exocyclic carbonyl group and are most likely affected by the shielding anisotropy of the carbonyl bond. In the *cis* form, the amide proton is perpendicular to the carbonyl double bond, which makes it shielded and leads to an upfield shift.

**Table 1:** Non‐exchangeable ^1^H and ^13^C chemical shifts (ppm) of *α*‐ and *β*‐GlcNAc in *trans* and *cis* amide forms.^[a]^

	H1	H2	H3	H4	H5	H6a/b		H2′
	C1	C2	C3	C4	C5	C6	C1′	C2′
*α‐trans*	5.20	3.86	3.75	3.47	3.84	3.83/3.78		2.04
	93.6	56.9	73.5	72.8	74.3	63.4	177.3	24.7
*α‐cis*	5.26	3.58	3.74	3.51	3.86	3.80/3.78		2.03
	94.3	60.7	74.1	n.d.	n.d.	n.d.	179.5	22.6
*β‐trans*	4.70	3.66	3.52	3.45	3.45	3.89/3.74		2.04
	97.7	59.5	76.7	72.6	78.6	63.5	177.5	24.9
*β‐cis*	4.70	3.30	3.47	n.d.	n.d.	3.92/3.75		2.03
	97.9	64.0	77.1	n.d.	n.d.	n.d.	180.4	23.0

[a] In 90 % H_2_O/10 % D_2_O at 25 °C; chemical shifts relative to DSS‐*d*_6_; n.d.=not determined.

**Table 2:** ^1^H/^15^N chemical shifts (ppm), one‐bond coupling constants (Hz), and temperature coefficients (*dδ/dT*, ppb/°C) of *trans* and *cis* amide groups in *α*‐ and *β*‐GlcNAc.^[a]^

	*δ*_N**H**_	*δ*_**N**H_	^1^ *J*_NH_	*dδ/dT*
*α‐trans*	8.10	123.6	(–) 92.8	−9.1
*α‐cis*	7.12	123.5	(–) 88.6	−6.9
*β‐trans*	8.19	122.9	(–) 91.5	−7.9
*β‐cis*	7.53	124.3	(–) 87.4	−8.5

[a] In 90 % H_2_O/10 % D_2_O, pH 6.7±0.1, at 25 °C; ppm relative to DSS‐*d*_6_.

The most affected carbon chemical shifts of the *cis* forms were those of C2, C1′ and C2′, with a downfield shift of C2 and C1′ resonances (∼4 ppm and 2–3 ppm, respectively) and an upfield shift (∼2 ppm) of C2′ resonances, compared to the *trans* forms (Table 1). These differences in ^13^C chemical shifts between *cis* and *trans* forms are consistent with previously determined rules for assignment of *Z* and *E* isomers of various sugar amides.^[27]^ The ^15^N chemical shift of the *β‐cis* isomer was shifted downfield (1.4 ppm) compared to the *β‐trans* isomer, but the ^15^N chemical shift of the *α*‐*cis* isomer was almost identical to the *α*‐*trans* isomer (Table 2).

Temperature coefficients (*dδ/dT*) of amide protons are used to indicate the existence of hydrogen bonds or reduced hydration in proteins^[28]^ and *dδ/dT* have also been measured from amide protons of GlcNAc and other *N*‐acetylated sugars.^[29]^ The temperature coefficients of *trans* and *cis* amide protons in *α*‐ and *β*‐ GlcNAc were determined over the range of 3 to 40 °C (Table 2). The values vary from −6.9 to −9.1 ppb/°C, which indicates that the amide protons do not form intramolecular hydrogen bonds to a large extent in aqueous solution, since temperature coefficients from −10 to −6 ppb/°C are usually indicative of the lack of intramolecular hydrogen bonds.^[30]^ However, the slightly less negative *dδ/dT* of *α‐cis* NH (−6.9 ppb/°C) compared to *α‐trans* NH (−9.1 ppb/°C) suggests that the *α‐cis* amide proton is less solvated, which could be due to steric effects, transient intramolecular hydrogen bonds or transient hydrogen bonds to water molecules.

The one‐bond coupling constant, ^1^ *J*_NH_, was determined from coupled ^1^H,^15^N‐HSQC spectra and showed lower values for the *cis* forms (87–89 Hz) than the *trans* forms (91–93 Hz). This is in accordance with peptide linkages, where ^1^ *J*_NH_ are typically in the range 92–94 Hz and 89–91 Hz for a *trans* and a *cis* conformation, respectively,^[31]^ The *α*‐*trans* NH showed a larger ^1^ *J*_NH_ than *β*‐*trans* NH, which is equivalent to previously reported ^1^ *J*_NH_ on GlcNAc.^[32]^

### Relative quantification of GlcNAc isomers

Relative quantification of the four GlcNAc isomers (*trans* and *cis* amides of the *α*‐ and *β*‐anomer, respectively) was obtained by integration of amide proton signals from 1D spectra of unlabeled GlcNAc over a temperature range from 3 to 40 °C (Figure S2). The GlcNAc *α*‐pyranoside dominates over the *β*‐pyranoside in their amide *trans* forms and it increases with temperature, which is consistent with earlier reports of 68 % *α*‐ and 32 % *β*‐pyranoside at 70 °C.^[33]^

The relative amount of amide *cis* forms increased with temperature. In contrast to the *trans* forms, the *β‐cis* isomer (0.6–1.2 %) is more populated than the *α‐cis* isomer (0.3–1.0 %) at all temperatures investigated. The equilibrium constants *K*_*trans/cis*_ were calculated and used to construct van't Hoff plots (see Table S1 and Figure S3), from which *ΔH°*_*cis→trans*_ were extracted and determined to −12.8 kJ/mol and −15.6 kJ/mol for the *α*‐ and the *β*‐pyranoside, respectively. *ΔS°*_*cis→trans*_ were determined to −3.9 J/K/mol for *α*‐pyranoside and −20.2 J/K/mol for *β*‐pyranoside. Amide *trans* forms in both *α*‐ and *β*‐pyranosides are enthalpically favored but entropically disfavored and *ΔG°*_*cis→trans*_ was calculated to −11.6 kJ/mol and −8.9 kJ/mol at 25 °C for the *α*‐ and the *β*‐pyranoside, respectively; thus amide *trans* forms are thermodynamically favored. Moreover, compared with *β*‐ pyranoside, *α*‐pyranoside has higher equilibrium constants *K*_*trans/cis*_ and more negative Gibbs free energy. The results are in the same order of magnitude as earlier investigations on GlcNAc methyl glycosides by Hu et al., except a more negative *ΔS°*_*cis→trans*_ for the *β*‐pyranoside (−20.2 J/K/mol) compared to the methyl glycoside (−2.9 J/K/mol).^[9]^

### NMR experiments for determination of GlcNAc *N*‐acetyl *J*‐coupling constants

**Figure 3:**
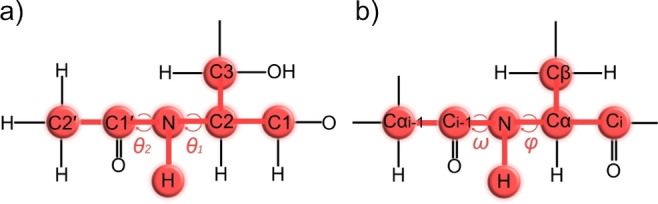
Comparison between a) the *N*‐acetyl group of GlcNAc and b) the protein backbone.

By utilizing the GlcNAc amide protons, we could use protein NMR experiments on uniformly ^13^C,^15^N‐labeled GlcNAc for determination of eight three‐bond *J*‐coupling constants within the *N*‐acetyl group. The *N*‐acetyl group of GlcNAc resembles the protein backbone with an amide function, Cα replaced by C2, and Cβ replaced by C3 (Figure 3). Since the protein backbone is a repeat of NH, Cα, and CO, but GlcNAc is not, CO_i‐1_ corresponds to GlcNAc C1′, whereas CO_i_ is replaced by C1, and Cα_i‐1_ is replaced by C2′. Thus, E.COSY‐type and *J*‐quantitative protein NMR experiments for coupling constants over the *ϕ* and *ω* backbone angles could be adapted and utilized for the *θ_1_* and *θ_2_* torsion angles of the *N*‐acetyl group.

Six vicinal coupling constants through the C2‐N bond are sensitive to the *θ_1_* torsion angle, ^3^ *J*_N_ ${}_{\underline{H}}$_,H2_, ^3^ *J*_N_ ${}_{\underline{H}}$_,C1_, ^3^ *J*_N_ ${}_{\underline{H}}$_,C3_, ^3^ *J*_H2,C1′_, ^3^ *J*_C1,C1′_, and ^3^ *J*_C3,C1′_, whereas two vicinal coupling constants can be extracted over the N‐CO bond, which are sensitive to the *θ_2_* torsion angle, namely ^3^ *J*_N_ ${}_{\underline{H}}$_,C2′_ and ^3^ *J*_C2,C2′._ The experiments are summarized in Table S2 and presented in detail in the experimental section. The standard deviation of the *J*‐couplings was typically 0.1 Hz for the *trans* forms and up to 0.3 Hz for the *cis* forms due to the low abundance of amide *cis* forms.

### *J*‐couplings sensitive to the *θ*_1_ angle (H2‐C2‐N‐H)

**Figure 4:**
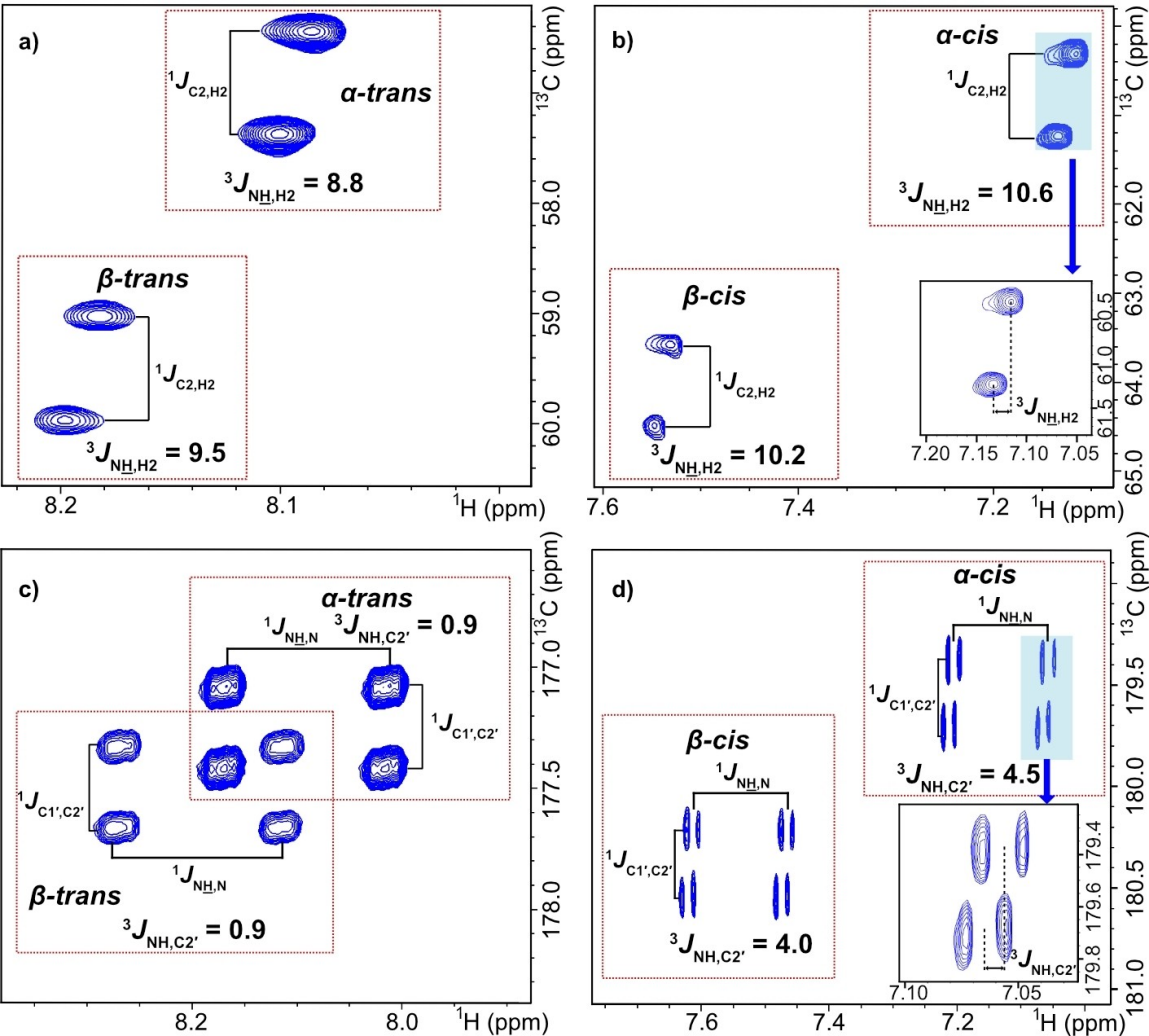
E.COSY spectra used to measure ^3^ *J*_N_ ${}_{\underline{H}}$_,H2_ of a) *trans* and b) *cis* forms, and ^3^ *J*_NH,C2′_ of c) *trans* and d) *cis* forms. The spectra in a) and b) were recorded with ^15^N decoupling whereas the spectra in c) and d) were recorded without decoupling.

The HNCA[HA]‐E.COSY experiment was used to determine ^3^ *J*_N_ ${}_{\underline{H}}$_,H2_, with selective ^13^C pulses and delays adjusted for GlcNAc (see experimental). The GlcNAc *trans* forms exhibited ^3^ *J*_N_ ${}_{\underline{H}}$_,H2_ of 8.8 Hz for the *α*‐anomer and 9.5 Hz for the *β*‐anomer (Figure 4a), which is equivalent to earlier reported values of 8.7–8.9 Hz for *α*‐GlcNAc and 9.1–9.8 Hz for *β*‐GlcNAc.[^17^, ^18^, ^32^] In addition, ^3^ *J*_N_ ${}_{\underline{H}}$_,H2_ of the GlcNAc *cis* forms could be measured, despite the much lower intensity. The *cis* forms exhibited 10.6 Hz for the *α*‐anomer and 10.2 Hz for the *β*‐anomer (Figure 4b), which is 1.8 Hz and 0.7 Hz larger compared to the *trans* form of *α*‐ and *β*‐GlcNAc, respectively. Earlier reported ^3^ *J*_N_ ${}_{\underline{H}}$_,H2_ from *cis* forms of *N*‐formyl substituted sugars in DMSO‐*d*_6_ have shown very similar values for *trans* and *cis* forms^[9]^ or a larger coupling constant of the *cis* form by 1.3 Hz.^[34]^

Karplus equations that were parametrized for ^3^ *J*_N_ ${}_{\underline{H}}$_,H2_ by Hu, et al.^[24]^ on GlcNAc model structures show maxima at 0° and 180°, corresponding to *θ_1_‐syn* and ‐*anti*, respectively. Separate Karplus equations were determined for *trans* and *cis* forms, where the *cis* forms have larger ^3^ *J*_N_ ${}_{\underline{H}}$_,H2_ than the *trans* forms of about 2 Hz.^[24]^ Our results are in agreement with these parametrizations, assuming that the *trans* and *cis* forms have the same conformation over the *θ_1_* linkage. By calculating the difference between observed ^3^ *J*_N_ ${}_{\underline{H}}$_,H2_ and the values from the parametrized Karplus equations over the whole span of torsion angles, a minimization plot is generated, where the global minimum corresponds to the most likely torsion angle (Figure S4a). However, for *α*‐ and *β*‐GlcNAc (*trans* as well as *cis* forms) minima are observed in two regions, around 0° and 180°, which cannot be distinguished. The calculated ^3^ *J*_N_ ${}_{\underline{H}}$_,H2_ is consistently higher in magnitude compared to experimental data (about 1–2 Hz), which has been attributed to the internal dynamics of the pyranose ring.^[17]^ This inconsistency between experimental and calculated ^3^ *J*_N_ ${}_{\underline{H}}$_,H2_ is in the same order of magnitude as the difference between the maxima in the Karplus curves corresponding to *syn* and *anti* conformation, which is 1.2–2.3 Hz depending on the parametrized equation. Consequently, the use of ^3^ *J*_N_ ${}_{\underline{H}}$_,H2_ alone to distinguish between *anti* and *syn* conformations is limited, as shown by our minimization plot, and additional *J*‐coupling constants are needed for the analysis.

The ^3^ *J*_H2,C1′_ coupling constant was measured from the (H)NCAHA(CO)‐E.COSY experiment to 2.8 and 3.4 Hz for the *trans* forms of *α*‐ and *β*‐GlcNAc, respectively (Table 3; Figure S5). This can be compared with earlier reported ^3^ *J*_H2,C1′_ of 3.1 for the methyl glycoside of *α*‐GlcNAc^[24]^ and 3.5 Hz for *β*‐GlcNAc.^[32]^ The *cis* forms showed similar ^3^ *J*_H2,C1′_ with 3.3 and 2.6 Hz for *α*‐ and *β*‐GlcNAc, respectively.

**Table 3:** ^3^ *J* and ^2^ *J* coupling constants (Hz) that are sensitive to the *θ_1_* and *θ_2_* angles in *α*‐ and *β*‐GlcNAc.^[a]^

	^3^ *J*_N_ ${}_{\underline{H}}$_,H2_	^3^ *J*_H2,C1′_	^3^ *J*_N_ ${}_{\underline{H}}$_,C1_	^3^ *J*_N_ ${}_{\underline{H}}$_,C3_	^3^ *J*_C1,C1′_	^3^ *J*_C3,C1′_	^3^ *J*_N_ ${}_{\underline{H}}$_,C2′_	^3^ *J*_C2,C2′_	^2^ *J*_C2,C1′_^[b]^
*α‐trans*	8.8±0.02	2.8±0.11	0.4±0.01	0.9±0.02	1.0±0.01	1.4±0.03	0.9±0.05	1.4±0.04	0.5±0.13
*α‐cis*	10.6±0.18	3.3±0.02	0.7±0.09	0.7±0.11	1.6±0.03	obs.^[c]^	4.5±0.11	<0.5^[d]^	1.1±0.31
*β‐trans*	9.5±0.01	3.4±0.04	0.6±0.02	0.8±0.01	1.0±0.23	1.0±0.04	0.9±0.06	1.7±0.06	0.9±0.03
*β‐cis*	10.2±0.26	2.6±0.23	0.7±0.06	0.9±0.24	obs.^[c]^	obs.^[c]^	4.0±0.07	<0.5^[d]^	1.2±0.20

[a] At 25 °C in 90 % H_2_O/10 % D_2_O. Data are presented from at least three measurements with ±one standard deviation. [b] Sign unknown. [c] Obscured signals. [d] Below the detection limit.

In contrast to ^3^ *J*_N_ ${}_{\underline{H}}$_,H2_, ^3^ *J*_H2,C1′_ values differ clearly between 0° and 180°, corresponding to *anti* and *syn* conformation, respectively. Calculated Karplus equations show much larger ^3^ *J*_H2,C1′_ at 180° (8.0–8.5 Hz) compared to 0° (4.0–4.4 Hz) and thus the observed ^3^ *J*_H2,C1′_ for both *trans* and *cis* forms are consistent with *θ_1_*‐*anti* rather than *θ_1_*‐*syn* conformation. However, due to the symmetrical shape of the Karplus curve, ^3^ *J*_H2,C1′_ alone cannot be used to determine torsion angles in‐between *anti* and *syn* conformation, which might be populated if the *N*‐acetyl group is involved in hydrogen bonding. For an accurate determination of the torsion angle, a set of several coupling constants is necessary.

The ^3^ *J*_N_ ${}_{\underline{H}}$_,C1_ and ^3^ *J*_N_ ${}_{\underline{H}}$_,C3_ coupling constants were measured from HNCA[CB]‐E.COSY experiments with off‐resonance carbon pulses on C3 and C1, respectively. Both *trans* and *cis* forms of *α*‐ and *β*‐GlcNAc exhibited ^3^ *J*_N_ ${}_{\underline{H}}$_,C1_ and ^3^ *J*_N_ ${}_{\underline{H}}$_,C3_ in the range 0.4–0.9 Hz (Table 3; Figure S5). This is consistent with an *anti* conformation, with calculated ^3^ *J*_N_ ${}_{\underline{H}}$_,C1_ and ^3^ *J*_N_ ${}_{\underline{H}}$_,C3_ of 0.8–1.2 Hz from parametrized Karplus equations.^[24]^ The same equations predict 1.7–2.8 Hz for *syn* conformations, with ^3^ *J*_N_ ${}_{\underline{H}}$_,C1_ slightly larger than ^3^ *J*_N_ ${}_{\underline{H}}$_,C3_. Since the amide proton in the *anti* conformation is in a *gauche* position to C1 and C3 (−60° and +60°, respectively), the ^3^ *J*_N_ ${}_{\underline{H}}$_,C1_ and ^3^ *J*_N_ ${}_{\underline{H}}$_,C3_ coupling constants are close to minima at ±90° in the Karplus curve and similarly the *syn* conformation, which corresponds to +120° and −120°, respectively, are close to the minima. However, if the *θ_1_* torsion angle deviates from the *anti* and *syn* conformations, ^3^ *J*_N_ ${}_{\underline{H}}$_,C1_ and ^3^ *J*_N_ ${}_{\underline{H}}$_,C3_ are predicted to be much larger, up to 6–9 Hz for *θ_1_* =±60° (corresponding to 180° in the C1−C2−N−H or C3−C2−N−H Karplus curve).

Finally, a spin‐echo difference CT‐HSQC experiment was used to determine the ^3^ *J*_C1,C1′_ and ^3^ *J*_C3,C1′_ coupling constants. The 2D spectrum is identical to a ^1^H,^13^C‐HSQC spectrum, but with intensities affected by the C−CO couplings in a *J*‐quantitative manner.^[35]^ Since GlcNAc C1 and C3 cross‐peaks were well resolved (at least for the *trans* forms), ^3^ *J*_C1,C1′_ and ^3^ *J*_C3,C1′_ could be measured from the same experiment (Table 3). However, the C3 cross‐peaks of the *cis* forms were obscured by the much more intense *trans* forms, which prevented the measurement of ^3^ *J*_C3,C1′_. Similarly, ^3^ *J*_C1,C1′_ of the *α*‐GlcNAc *cis* form could not be determined due to the overlap of the C1 cross‐peak with the *trans* form. The cross‐peak from the *trans β*‐GlcNAc C1 was close to the residual water signal and therefore the standard deviation for this measurement was significantly higher (0.23 Hz).

All ^3^ *J*_C1,C1′_ and ^3^ *J*_C3,C1′_ were in the range 1.0–1.6 Hz, which is consistent with previously reported data.[^9^, ^32^] According to parametrized Karplus equations,^[24]^ a *θ_1_*‐*anti* conformation corresponds to ^3^ *J*_C1,C1′_ and ^3^ *J*_C3,C1′_ in the range 1.1–1.5 Hz, whereas a *syn* conformation corresponds to ^3^ *J*_C1,C1′_ and ^3^ *J*_C3,C1′_ close to zero. Thus, the obtained *J* couplings are consistent with a *θ_1_*‐*anti* conformation. As for ^3^ *J*_N_ ${}_{\underline{H}}$_,C1_ and ^3^ *J*_N_ ${}_{\underline{H}}$_,C3_, the orientation of C1 and C3 in relation to the amide proton (and C1′) implies that ^3^ *J*_C1,C1′_ and ^3^ *J*_C3,C1′_ are close to the minima at ±90° in the *θ_1_*‐*anti* and *syn* conformations. However, deviations from *θ_1_*‐*anti* and *syn* conformations would result in larger ^3^ *J*_C1,C1′_ and ^3^ *J*_C3,C1′_ of up to 3–4.5 Hz.

Other C−C couplings involving C1′ could also be measured using the spin‐echo difference CT‐HSQC experiment, including the two‐bond coupling ^2^ *J*_C2,C1′_, which was in the range of 0.5–1.2 Hz (Table 3). This is in accordance with earlier reports of 0.8–1.1 Hz,^[9]^ but there is no obvious correlation between ^2^ *J*_C2,C1′_ and the *θ_1_* or *θ_2_* torsion angle conformation.

### *J*‐couplings sensitive to the *θ*_2_ angle (C2−N−C1′−C2′)

^3^ *J*_N_ ${}_{\underline{H}}$_,C2′_ was measured from the HNCO[CA]‐E.COSY experiment with selective pulses on the methyl carbon rather than Cα (see experimental). A clear difference was observed between GlcNAc *trans* conformations, with ^3^ *J*_N_ ${}_{\underline{H}}$_,C2′_ of 0.9 Hz for both anomers, and GlcNAc *cis* conformations, with ^3^ *J*_N_ ${}_{\underline{H}}$_,C2′_ of 4.5 and 4.0 Hz for *α*‐ and *β*‐GlcNAc, respectively (Figure 4c and d). This is close to predicted values from parametrized Karplus equations, which are 1.0 Hz for a *trans* conformation and 4.8 Hz for a *cis* conformation.^[24]^ Thus, ^3^ *J*_N_ ${}_{\underline{H}}$_,C2′_ can be successfully used to distinguish between *trans* and *cis* amide conformation and, in the case of GlcNAc, it confirms the *cis* conformation of the minor GlcNAc species.

^3^ *J*_C2,C2′_ was measured by a *J*‐quantitative long‐range (H)C(C)H experiment, which can be used to measure several *J*_CC_ couplings within the ring or, in the case of oligosaccharides, over the glycosidic linkage.^[36]^ The *trans* forms showed ^3^ *J*_C2,C2′_ of 1.4 and 1.7 Hz for *α*‐ and *β*‐GlcNAc, respectively (Table 3; Figure S6), but the *cis* forms were below the detection limit (<0.5 Hz). These values are in agreement with previously measured ^3^ *J*_C2,C2′_ of 1.6–1.8 Hz from GlcNAc.^[9]^ Parametrized Karplus equations have shown that ^3^ *J*_C2,C2′_ is affected by both the *θ_1_* and the *θ*_2_ angle.^[24]^ *Trans* forms are predicted to 3.3 Hz in *θ_1_*‐*syn* conformation and 1.5 Hz in the *θ_1_*‐*anti* conformation, whereas *cis* forms are predicted to have ^3^ *J*_C2,C2′_ close to 0 Hz in both *θ_1_*‐*syn* and *anti* conformations. Thus, our observed values for the *trans* forms are equivalent to the *θ_1_*‐*anti* conformation and ^3^ *J*_C2,C2′_ for the *cis* forms are not detected because it is probably close to 0 Hz.

### The overall geometry of the *N*‐acetyl group

In order to generate single‐state models for the torsion angles *θ_1_* (H2−C2−N−H) and *θ_2_* (C2−N−C1′−C2′), absolute differences were calculated between our experimental results (Table 3) and the values from parametrized Karplus equations (Table S3). The differences were then normalized to a common torsion angle (*θ_1_* and *θ_2_*) and root‐mean‐square deviations (RMSD) were calculated over the full range of torsion angles for the full ensemble of coupling constants (six coupling constants related to *θ_1_* and two coupling constants related to *θ_2_*).

**Figure 5:**
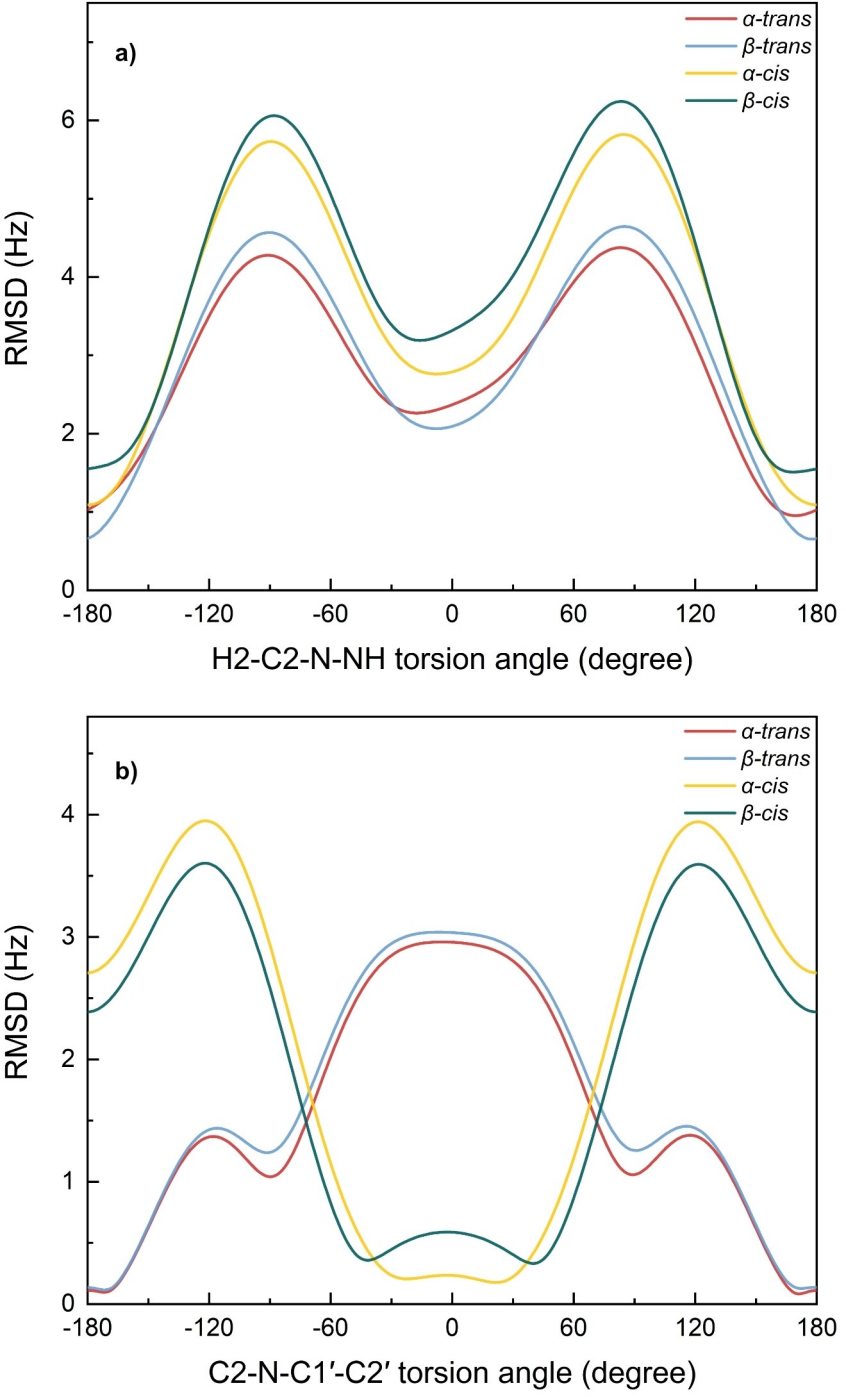
The overall single‐state plots of a) RMSD versus H2−C2−N−NH torsion angle using all six coupling constants that are sensitive to *θ_1_* and b) RMSD versus C2−N−C1′−C2′ torsion angle using ^3^ *J*_C2,C2′_ and ^3^ *J*_NH,C2′_ that are sensitive to *θ_2_*.

The plots of RMSD versus torsion angles H2−C2−N−H and C2−N−C1′−C2′ are presented in Figure 5. For the *θ_1_* torsion angle (Figure 5a) the global minimum, corresponding to the smallest deviation between calculated and experimental *J*‐couplings, can be found in the region from 168° to 180° (*anti* conformation) with *α‐trans* at (+170±10)°, *β*‐*trans* at (+178±6)°, *α*‐*cis* at (180±10)° and *β‐cis* at (+168±19)°. Other local minima are located in the region near 0° (*syn* conformation), but with RMSD that are about 1.5 Hz larger. The results are consistent with earlier reports on *θ_2_*‐*trans* forms of GlcNAc, where a similar treatment of *J*‐couplings on the *α*‐GlcNAc methyl glycoside was used to identify a global RMSD minimum at +160°,^[24]^ whereas MD simulations found an average *θ_1_* angle at +165° for *α*‐GlcNAc^[8]^ or +161° and 180° for *α*‐ and *β*‐GlcNAc, respectively.^[17]^

The *J*‐couplings were also subdivided into four groups to obtain individual RMSD plots: Group 1 with ^3^ *J*_N_ ${}_{\underline{H}}$_,H2_ and ^3^ *J*_H2,C1′_; Group 2 with ^3^ *J*_N_ ${}_{\underline{H}}$_,C1_ and ^3^ *J*_N_ ${}_{\underline{H}}$_,C3_; Group 3 with ^3^ *J*_C1,C1′_ and ^3^ *J*_C3,C1′_; and Group 4 with ^3^ *J*_N_ ${}_{\underline{H}}$_,C1_, ^3^ *J*_N_ ${}_{\underline{H}}$_,C3_, ^3^ *J*_N_ ${}_{\underline{H}}$_,H2_ and ^3^ *J*_H2,C1′_ (Figure S7). The *anti* and *syn* conformation can be most clearly distinguished from Group 1, where the difference in RMSD between the two rotamers is about 3 Hz for all the GlcNAc forms. Also, Group 2 can be used to distinguish between *anti* and *syn* conformation, though with a smaller difference in RMSD of about 1 Hz. Group 3, on the other hand, cannot be used to find the correct conformation, whereas Group 4 is almost identical to the plot of all six *J*‐couplings. In conclusion, ^3^ *J*_N_ ${}_{\underline{H}}$_,H2_ and ^3^ *J*_H2,C1′_ are enough to distinguish between *anti* and *syn* conformation. To determine the exact *θ_1_* angle, the addition of ^3^ *J*_N_ ${}_{\underline{H}}$_,C1_ and ^3^ *J*_N_ ${}_{\underline{H}}$_,C3_ is necessary, since ^3^ *J*_N_ ${}_{\underline{H}}$_,H2_ and ^3^ *J*_H2,C1′_ cannot be used to differentiate between positive and negative *θ_1_* due to the symmetry of the Karplus curves. Finally, the entire set of *J*‐couplings may be necessary in the rare case of *θ_1_* around ±90° (in between *anti* and *syn* conformation), where ^3^ *J*_N_ ${}_{\underline{H}}$_,H2_ and ^3^ *J*_H2,C1′_ are close to 0 Hz and ^3^ *J*_N_ ${}_{\underline{H}}$_,C1_ and ^3^ *J*_N_ ${}_{\underline{H}}$_,C3_ are similar for +90° and −90°.

In order to investigate minor contribution of *syn* conformation, a two‐state model for the *θ*_1_ torsion angle was generated (Figure S8). The torsion angle was fixed at 180° (*anti*) and 0° (*syn*) and the relative contribution of the two conformations was optimized by minimizing the RMSD between experimental and calculated *J*‐couplings, as in the single‐state model. For *α*‐*trans*, *α*‐*cis*, and *β*‐*cis* forms, the lowest RMSD was found at 100 % *anti* conformation, but for *β*‐*trans* a small contribution of *syn* conformation was obtained (2 % *syn*, 98 % *anti*). Interestingly, MD simulations on GlcNAc *trans* forms have shown a similar tendency with 100 % *anti* conformation of *α*‐GlcNAc, but 87 % *anti* and 13 % *syn* conformation of *β*‐GlcNAc.^[17]^

A single‐state model of the *θ_2_* torsion angle exhibited RMSD plots (Figure 5b) with global minima located as expected, close to 180° for *trans* conformation and close to 0° for *cis* conformation. The minima of the *trans* amides were located at ±172°, but due to an almost flat RMSD curve around 180°, it is not possible to distinguish between +172° and −172° or to exclude a planar 180° conformation. For the *cis* forms, ^3^ *J*_C2,C2′_ was set to 0 Hz (<0.5 Hz from experiments) and the exact torsion angles could not be determined accurately due to broad minima around 0°. Overall, ^3^ *J*_N_ ${}_{\underline{H}}$_,C2′_ is enough to differentiate between *trans* and *cis* amide conformation (Figure S4b), whereas both coupling constants (^3^ *J*_N_ ${}_{\underline{H}}$_,C2′_ and ^3^ *J*_C2,C2′_) are necessary to determine the *θ_2_* torsion angle in detail.

It is noteworthy that the RMSD in the global minima of the single‐state models are considerable for the *θ_1_* torsion angle (0.6–1.5 Hz), but much lower for the *θ_2_* torsion angle (0.1–0.3 Hz), despite up to six *J*‐couplings used for *θ_1_* and only two for *θ_2_*. This could be explained by 1) contribution from a less populated *θ_1_*‐*syn* conformation, 2) experimental errors, and 3) limitations of the parametrized Karplus equations. However, our two‐state model incorporating both *anti* and *syn* conformation did not improve the RMSD since the *syn* conformation was only populated by 0–2 %. Experimental errors of the measured coupling constants probably contribute to the RMSD, but the use of several different coupling constants with different torsion angle dependencies should minimize this effect so that the global minimum is detected with higher accuracy. Finally, the parametrized Karplus equations derived for GlcNAc model structures may have limitations due to the use of an implicit water model, which may not accurately mimic the real water‐solute interactions.^[24]^ It should also be mentioned that the dynamics of the GlcNAc ring is not included in the models and could affect the *J*‐couplings.^[17]^ Actually, the two most deviating *J*‐couplings, ^3^ *J*_N_ ${}_{\underline{H}}$_,H2_ and ^3^ *J*_H2,C1′_, both involve H2, which is probably most affected by ring puckering and other dynamic effects.

The preference for the *α*‐anomeric configuration of GlcNAc, in contrast to glucose, where the *β*‐anomeric configuration is preferred, is not well understood. It has been hypothesized that hydrogen bonding between NH and O1 would stabilize the *α*‐pyranoside^[37]^ and that unfavorable electrostatic interaction between O1 and C1′O is minimized in the *α*‐pyranoside.^[38]^ From our data, there is no indication of direct NH−O1 hydrogen bonding in *α*‐GlcNAc, based on the NH temperature coefficients showing similar values for the two anomers, and the average *θ_1_* torsion angle, which predicts that the amide proton is pointing away from O1. However, the interaction between NH and O1 through water bridges is likely to be significant in *α*‐GlcNAc as earlier reported from MD simulations.^[17]^ Such interaction may stabilize the *α*‐GlcNAc *θ_1_‐anti* conformation, which could explain why the *α*‐GlcNAc *θ_1_‐syn* conformation was not detected by our two‐state model. In addition, the *θ_1_‐syn* conformation of *α*‐GlcNAc exhibits a possibly unfavorable steric interaction between the axial O1 and the carbonyl oxygen. The *β*‐pyranoside, on the other hand, showed a more diverse appearance with a small fraction of *θ_1_‐syn* conformation and a larger proportion of amide *cis* conformation, compared to the *α*‐pyranoside. Given that *β*‐GlcNAc *θ_1_‐syn* conformation is free from steric hindrance, this conformation is expected in minor amounts. The reason for a larger proportion of *cis* conformation in *β*‐GlcNAc is less evident, but the more negative Δ*S*°_*cis*→*trans*_ for *β*‐GlcNAc, compared to *α*‐GlcNAc, suggests that differences in the water‐solute interactions of the two anomers might be an important factor for the *cis*‐*trans* equilibria.

## Conclusions

In this study, we report the first identification of NH protons of the amide *cis* forms of *α*‐ and *β*‐GlcNAc by NMR spectroscopy. The *cis* amide protons were distinguished by upfield chemical shifts and smaller ^1^ *J*_NH_, compared to the *trans* forms. The chemical shifts of the *cis* forms in aqueous solution were assigned and the temperature coefficients (*dδ/dT*) of amide protons were measured. The amide proton signals were also used for the relative quantification of each isomer over a temperature range from 3 °C to 40 °C, showing that the *β‐cis* isomer (0.6–1.2 %) is more populated than the *α*‐*cis* isomer (0.3–1.0 %) with an increasing amount of *cis* forms at higher temperatures. Thermodynamic analysis showed that amide *trans* forms in both *α* and *β* pyranosides are enthalpically favored but entropically disfavored, which might be due to solvation effects.

The amide protons of GlcNAc were further utilized by adapting E.COSY and *J*‐quantitative protein NMR experiments for ^13^C,^15^N‐labelled GlcNAc to measure eight *J*‐couplings along the *N*‐acetyl side chain. The experimental data were compared with parametrized Karplus equations and the full set of *J*‐couplings was used to identify the most probable conformation. For both *trans* and *cis* amide forms, the orientation between H2 and NH was determined as *anti* with a torsion angle (*θ_1_*) of +168°–180°. A two‐state model was also generated, showing no contribution of the *syn* conformation except for the *trans* form of *β*‐GlcNAc, where 2 % of *θ_1_*‐*syn* conformation was predicted. The orientation of the amide linkage for the *trans* and *cis* forms was confirmed by two *J*‐couplings that are sensitive to the *θ_2_* torsion angle. The ^3^ *J*_N_ ${}_{\underline{H}}$_,C2′_ coupling constant was found to be particularly useful to distinguish between amide *cis* and *trans* conformation.

The larger proportion of *cis* amide and *θ_1_*‐*syn* conformation in *β*‐GlcNAc suggests higher flexibility of the *N*‐acetyl group of *β*‐GlcNAc compared to *α*‐GlcNAc. This fact is of special interest in studies on glycans and glycoconjugates containing *β*‐GlcNAc, such as chitin, hyaluronic acid and *N*‐ and *O*‐glycosylated proteins. The presence of *cis* amide and/or *θ_1_*‐*syn* conformation in these biologically important molecules may have crucial effects on the overall conformation and the physicochemical properties. The tailored *J*‐coupling NMR experiments utilized herein for *N*‐acetyl conformation analysis provide tools for detecting these forms in complex glycoconjugates with isotopic labeling.

## Experimental Section

*Syn* and *anti* conformations are related to the torsion angle *θ_1_* (H2−C2−N−H) so that *syn* and *anti* conformation correspond to 0° and 180°, respectively. *Cis* and *trans* conformations are related to the torsion angle *θ_2_* (C2−N−C1′−C2′), so that *cis* and *trans* conformation correspond to 0° and 180°, respectively (Scheme 2). See Table S3 for relations to other torsion angles within the *N*‐acetyl group.

**Sample preparation**: Unlabeled *N*‐acetyl‐D‐glucosamine (CAS.RN 7512‐17‐6) was purchased from Sigma‐Aldrich and was dissolved in 90 % H_2_O/10 % D_2_O (600 μL) to obtain a 57 mM solution. The pH of the sample was adjusted to 7.0 with HCl or NaOH solutions and DSS‐*d*_6_ (0.5 mM) was added as a chemical shift reference (*δ*_H_ 0.00 ppm). The sample was transferred into a 5 mm NMR sample tube for further analysis.

*N*‐[1, 2‐^13^C_2_] acetyl‐D‐[UL‐^13^C_6_; ^15^N] glucosamine (CAS.RN 478529‐44‐1) was purchased from Omicron Biochemicals, Inc. (South Bend, IN, USA) and was used without further purification. UL‐GlcNAc (1 mg) was dissolved in 90 % H_2_O/10 % D_2_O (160 μL) to obtain a 27 mM solution. The pH of the sample was adjusted to 6.7 with HCl or NaOH solutions. The sample was transferred into a 3 mm NMR sample tube for further analysis.

**NMR Spectroscopy**: NMR spectra were recorded at 25 °C, unless otherwise stated, on a Bruker Avance III 600 MHz spectrometer using a 5 mm ^1^H/^13^C/^15^N/^31^P inverse detection CryoProbe equipped with a z‐gradient. Parameter settings for the NMR experiments are summarized in Table S4. NMR spectra were processed with TopSpin 4.0.6 (Bruker).

^1^H NMR experiments with water suppression using excitation sculpting were recorded on the unlabeled GlcNAc sample at 5 °C intervals from 3 to 40 °C to determine amide proton temperature coefficients and the percentages of *cis* and *trans* amide. Automatic phase correction, baseline correction and linear prediction were conducted prior to peak integration of *cis* and *trans* amide protons. For each temperature, at least three spectra were recorded and the average values were calculated. The temperature was controlled with 4 % MeOH in methanol‐*d*_4_ and was within ±1 °C.

1D EXSY experiments were performed on unlabeled GlcNAc to detect the exchange between *cis* and *trans* conformation. A Gaussian 180° pulse (80 ms) was used for excitation of selected amide protons and excitation sculpting was used for water suppression.

For resonance assignments of GlcNAc *cis* and *trans* forms, the UL‐GlcNAc sample was used for ^1^H,^13^C‐constant time (CT)‐HSQC, ^1^H,^15^N‐HSQC, ^1^H,^15^N‐HSQC‐TOCSY, HNCACB, and (H)C(C)H‐TOCSY experiments. In addition, 1D and 2D TOCSY experiments with selective excitation^[39]^ of the *cis* form amide protons were recorded on unlabeled GlcNAc to visualize the ^1^H spin systems of the *cis* forms without spectral overlap with the much more abundant *trans* forms.

The NMR experiments for spin‐spin coupling constants are described in detail in the Supporting Information with changes of delays and carbon pulses compared to the default protein NMR experiments. All the experiments were recorded as 2D experiments since the four GlcNAc amide proton signals could be resolved in the 1D ^1^H spectrum.

The homonuclear coupling constant ^3^ *J*_N_ ${}_{\underline{H}}$_,H2_ was extracted from the HNCA[HA]‐E.COSY experiment,^[40]^ where H2 becomes the passive spin with ^1^ *J*_C2,H2_ of about 145 Hz as associated coupling. Selective carbon pulses were adjusted for C2 and C1′, instead of Cα and CO.

^3^ *J*_N_ ${}_{\underline{H}}$_,C1_ and ^3^ *J*_N_ ${}_{\underline{H}}$_,C3_ were measured from HNCA[CB]‐E.COSY experiments,^[41]^ with C1 and C3, respectively, becoming the passive spin instead of Cβ. The two coupling constants were differentiated by carbon pulses selective on either C3 to yield ^3^ *J*_N_ ${}_{\underline{H}}$_,C1_ or on C1 to yield ^3^ *J*_N_ ${}_{\underline{H}}$_,C3_, instead of CO pulses in the original experiment. Fortunately, the C1, C2, and C3 carbons of GlcNAc resonate in different spectral regions (at about 95, 60 and 75 ppm, see Table 1), which allows selective pulses that can be distinguished between these different carbons. The associated coupling in the indirect dimension are ^1^ *J*_C1,C2_ and ^1^ *J*_C2,C3_ of about 45 and 35 Hz, respectively, which makes them easily resolved.

The ^3^ *J*_H2,C1′_ coupling constant was measured from the (H)NCAHA(CO)‐E.COSY experiment,^[42]^ where C1′ becomes the passive spin with ^1^ *J*_N,C1′_ of about 15 Hz as associated coupling.

The homonuclear carbon coupling constants, ^3^ *J*_C1,C1′_, ^2^ *J*_C2,C1′_, and ^3^ *J*_C3,C1′_, were measured from the spin‐echo difference CT‐HSQC experiment.^[35]^ The experiment is run in an interleaved manner, between a reference spectrum that equals a normal CT‐HSQC and the second experiment where ^3^ *J*_C1,C1′_ couplings are active during the constant time period. The coupling constants are calculated from the relation between the cross‐peak intensities in the two spectra in a *J*‐quantitative manner.

^3^ *J*_N_ ${}_{\underline{H}}$_,C2′_ was measured from a HNCO[CA]‐E.COSY experiment,^[43]^ where the methyl carbon becomes the passive spin with the associated coupling ^1^ *J*_C1′,C2′_ of about 50 Hz. Carbon pulses selective on Cα were exchanged to C2′ at 23–25 ppm, which resonate in a region that is clearly distinguished from all other GlcNAc carbons.

A *J*‐quantitative long‐range (H)C(C)H experiment was used to measure ^3^ *J*_C2,C2′_.^[44]^ It is a COSY‐type experiment where the correlation is related to the size of the *J*_CC_ coupling. Since C2′ chemical shifts are different from other carbons, the correlations to C2 could be resolved.

## Conflict of interest

The authors declare no conflict of interest.

1

## Supporting information

As a service to our authors and readers, this journal provides supporting information supplied by the authors. Such materials are peer reviewed and may be re‐organized for online delivery, but are not copy‐edited or typeset. Technical support issues arising from supporting information (other than missing files) should be addressed to the authors.

Supporting Information

## Data Availability

The data that support the findings of this study are available in the supplementary material of this article.
