# Supplementary material for: Determination of Amide cis/trans Isomers in N‐Acetyl‐d‐glucosamine: Tailored NMR Analysis of the N‐Acetyl Group Conformation
Source: Chembiochem. 2022 Jul 11;23(17):e202200338. doi: 10.1002/cbic.202200338 (PMC9541821; doi:10.1002/cbic.202200338)
Supplement: Supplementary file 1 — Supporting Information [file CBIC-23-0-s001.pdf]

ChemBioChem

Supporting Information

Determination of Amide *cis*/*trans* Isomers in *N*-Acetyl-D-glucosamine: Tailored NMR Analysis of the *N*-Acetyl Group Conformation

Yan Xue and Gustav Nestor*

1. Supplementary Methods
   Spin-spin coupling constant experiments S2
2. Supplementary Figures
   Figure S1 .... S4
   Figure S2 .... S4
   Figure S3 .... S4
   Figure S4 .... S5
   Figure S5 .... S6
   Figure S6 .... S7
   Figure S7 .... S7
   Figure S8 .... S8
3. Supplementary Tables
   Table S1 .... S9
   Table S2 .... S9
   Table S3 .... S10
   Table S4 .... S11
4. References S12

Supplementary Methods

Spin-spin coupling constant experiments

See also Table S4 for additional parameters.

HNCA[HA]-E.COSY ($^{3}J_{N\underline{H},H2}$)

The carrier frequency was positioned at 56 ppm (C2) for ^{13}C and 123 ppm for ^{15}N. Off-resonance 180° shaped carbon pulses were positioned at 83 ppm to cover C1 and C3, but not C2. Pulse durations were 800 µs for off-resonance 180° Q3-surbop, 1.4 ms for on-resonance 180° Q3-surbop and 1.5 ms for 90° Q5-sebop pulses. Simultaneous decoupling of C2 and C1′ was achieved with an MLEV-16 expansion of Q3 pulses of 768 µs length. ^{15}N was decoupled using the GARP decoupling sequence. The delays were set as follows: $\Delta/2$ = 2.3 ms, Δ' = 2.7 ms, $\tau/2$ = 13 ms, $\tau'/2$ = 11.1 ms, and ε = 1.2 ms. Delay notations are referred to Weisemann et al. (1994).[1]

HNCA[CB]-E.COSY ($^{3}J_{N\underline{H},C1}$ and $^{3}J_{N\underline{H},C3}$)

Off-resonance 180° shaped carbon pulses were positioned at C3 (76 ppm) and C1 (95 ppm) for measuring $^{3}J_{N\underline{H},C1}$ and $^{3}J_{N\underline{H},C3}$, respectively. On-resonance shaped pulses were positioned at C2 (56 ppm). To make the pulses selective on either C1, C2, or C3, the pulse duration was set to 1.4 ms for 180° Q3-surbop and 1.5 ms for 90° Q5-sebop pulses. The carrier for the ^{15}N pulses was positioned at 123 ppm and ^{15}N GARP4 decoupling was applied during the acquisition with 567 µs pulse duration to allow for a long acquisition time (854 ms). Delay durations were τ = 2.3 ms, Δ = 5.4 ms, and T = 13 ms. Delay notations are referred to Wang and Bax (1996).[2]

(H)NCAHA(CO)-E.COSY ($^{3}J_{H2,C1'}$)

The carrier frequency was positioned at 55 ppm (C2) for ^{13}C and 123.2 ppm for ^{15}N. Off-resonance 180° shaped carbon pulses were positioned at C1′ (180 ppm) and the 180° pulse on aliphatic carbons during the ^{13}C constant-time evolution period was centered at 65 ppm. Pulse durations were 270 µs for 180° Q3-surbop and 350 µs for 90° Q5-sebop pulses. No ^{13}C or ^{15}N decoupling was used during the acquisition. Delay durations were τ = 2.3 ms, η = 5.4 ms, $T_N/2$ = 15.5 ms, $T_C/2$ = 11.1 ms, κ = 2.1 ms, and δ = 1.7 ms. Delay notations are referred to Löhr and Rüterjans (1995).[3]

Spin-echo difference CT-HSQC ($^{3}J_{C1,C1'}$, and $^{3}J_{C3,C1'}$)

The carrier frequency was positioned at 80 ppm for ^{13}C and 117 ppm for ^{15}N. Off-resonance 180° shaped carbon pulses were positioned at C1′ (178 ppm) and the pulse durations for shaped carbon pulses were 270 µs (180° Q3-surbop). ^{13}C was decoupled using the GARP decoupling sequence. The delays were set as follows: τ = 1.7 ms and T = 27 ms, where the constant-time delay ($2T = n/^{1}J_{CC}$) was a compromise between $^{1}J_{C1,C2}$ = 45 Hz and $^{1}J_{C2,C3}$ = 35 Hz.

HNCO[CA]-E.COSY ($^{3}J_{N\underline{H},C2'}$)

The carrier frequency was positioned at 174.8 ppm (C1′) for ^{13}C and 123 ppm for ^{15}N. Off-resonance 180° shaped carbon pulses were positioned close to C2′ (10 ppm). Pulse durations were 500 µs for off-resonance 180° Q3-surbop, 270 µs for on-resonance 180° Q3-surbop and 350 µs for 90° Q5-sebop pulses. No ^{13}C or ^{15}N decoupling was used during the acquisition. The delays were set according to the default values in the pulse program.

***J*-quantitative long-range (H)C(C)H ($^3J_{C2,C2'}$)**

The carrier frequency was positioned at 98 ppm for ^{13}C and 123 ppm for ^{15}N. Off-resonance 180° Q3-surbop carbon pulses were positioned at C1′ (180 ppm) and 180° Q3-surbop pulses for aliphatic carbons were positioned at 63 ppm, both pulses with a duration of 270 μs. In addition, on-resonance ^{13}C inversion was achieved with a 60 kHz smoothed Chirp pulse (0.5 ms, 20.1% smoothing). ^{13}C was decoupled using the GARP decoupling sequence. The delays were set as follows: τ = 1.6 ms and T = 22.2 ms.

Supplementary Figures

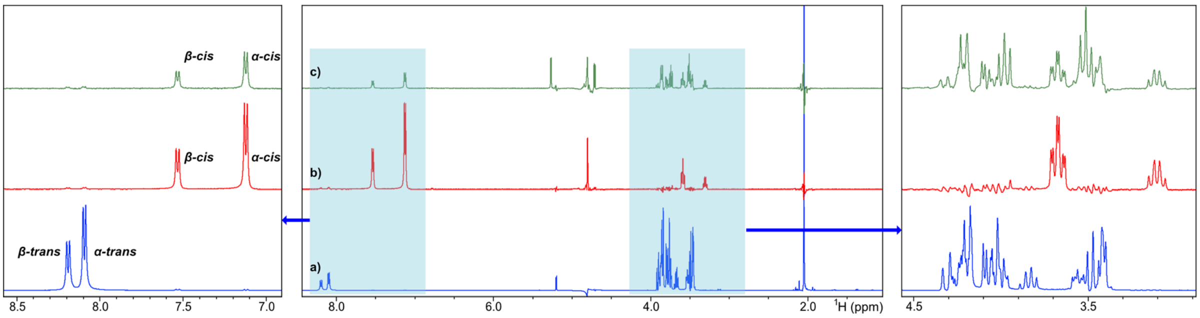

Figure S1. ^{1}H NMR spectrum with excitation sculpting a) and bandselective 1D TOCSY spectra with excitation of the *cis* amide protons and a mixing time of 20 ms b) and 120 ms c).

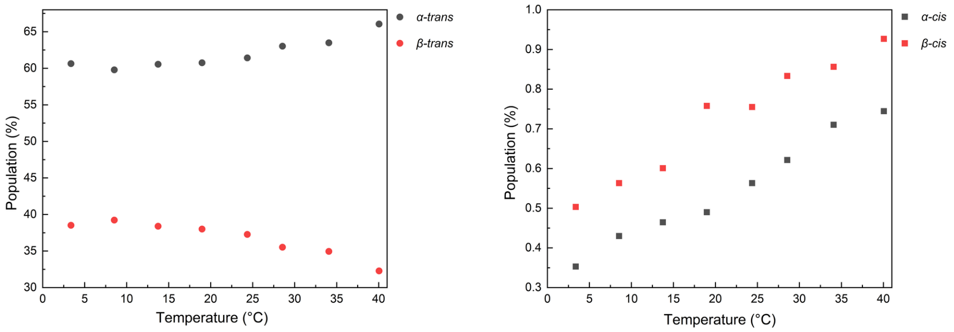

Figure S2. The relative concentration of *trans* and *cis* amide of *α*- and *β*-GlcNAc over a temperature range from 3 to 40 °C.

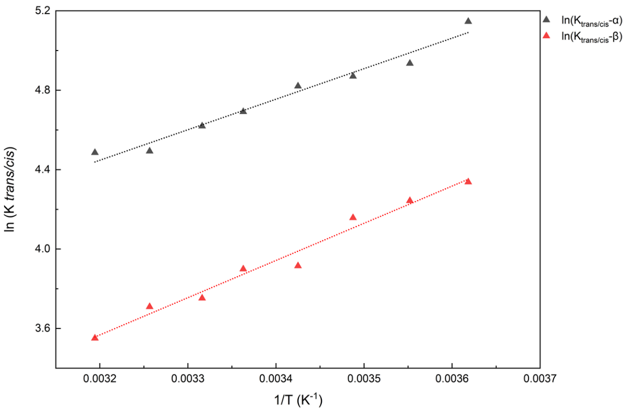

Figure S3. Van't Hoff plots for *α*- (black triangles) and *β*-GlcNAc (red triangles) using data from **Table SI 1**. $\Delta H^\circ_{cis\rightarrow trans}$, $\Delta S^\circ_{cis\rightarrow trans}$ and $\Delta G^\circ_{cis\rightarrow trans}$ values for the conversion of *cis* to *trans* amide in *α*- and *β*-pyranosides were determined (see text).

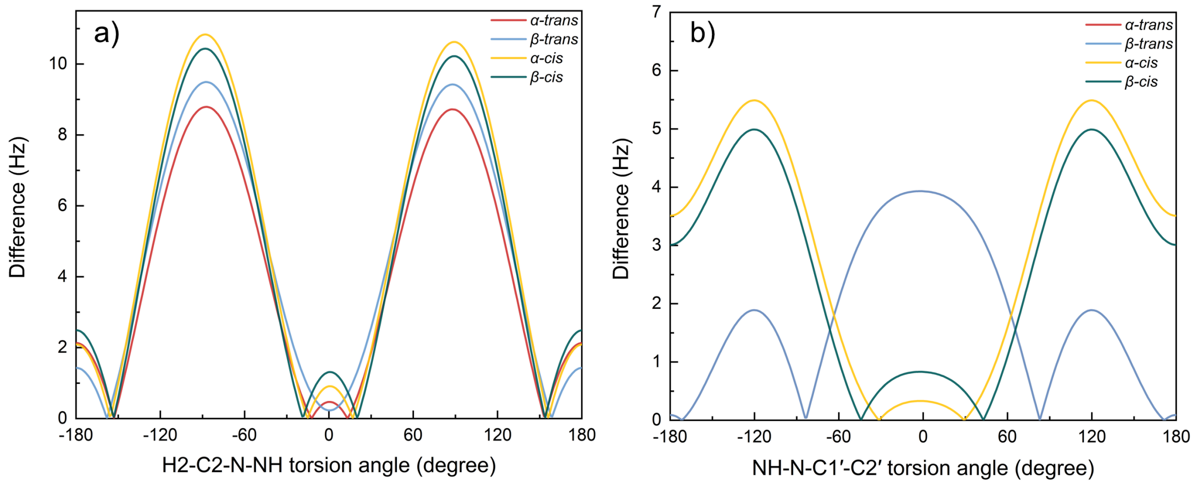

Figure S4. The difference between the experimental value of a) $^{3}J_{N\underline{H},H2}$ and b) $^{3}J_{N\underline{H},C2'}$ and the calculated values from the Karplus equations. In b), the curves for *α-trans* and *β-trans* are identical since the experimental values were the same (0.9 Hz).

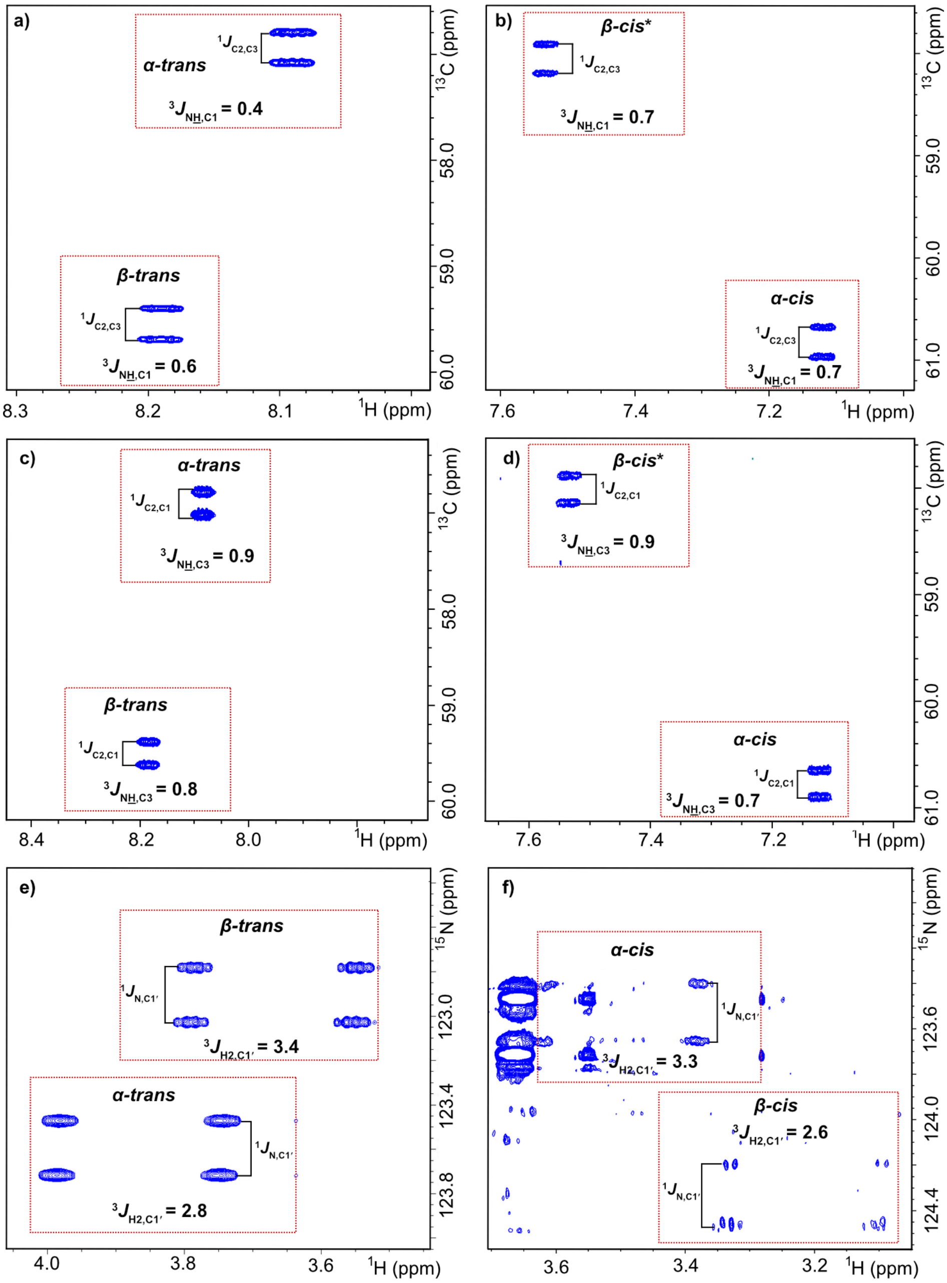

Figure S5. E.COSY spectra used to measure $^3J_{N\underline{H},C1}$ (a and b), $^3J_{N\underline{H},C3}$ (c and d), and $^3J_{H2,C1'}$ (e and f). Cross-peaks that are folded in the spectra are indicated by an asterisk (*).

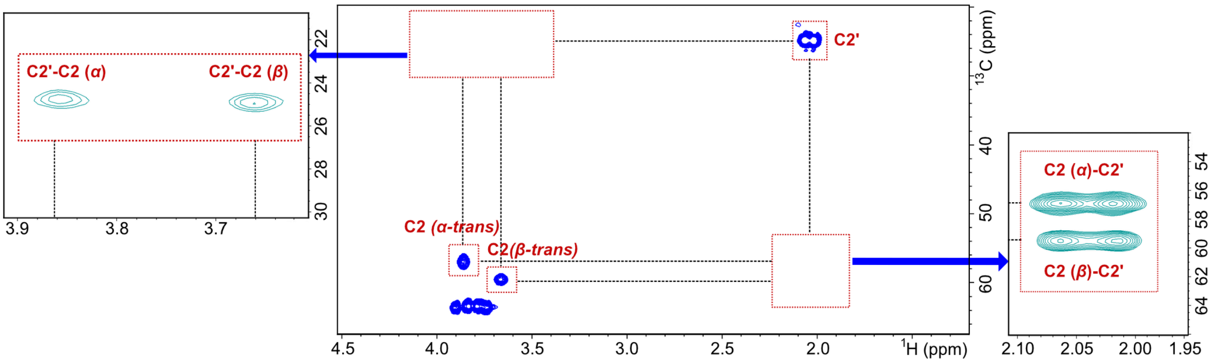

Figure S6. *J*-quantitative long-range (H)C(C)H spectrum used to measure $^3J_{C2,C2'}$. The *J*-couplings were measured from the cross-peaks corresponding to C2/H2′ (f1/f2).

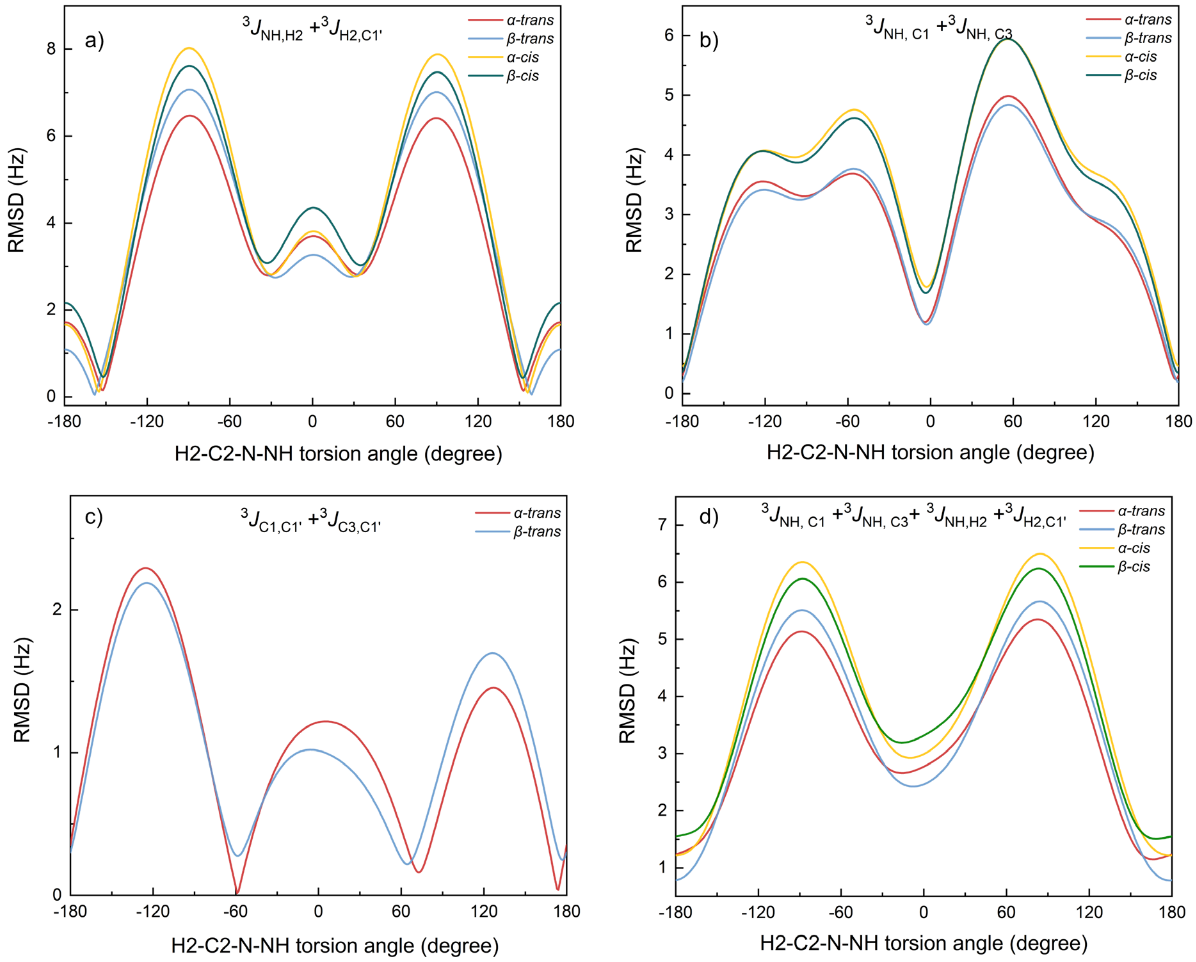

Figure S7. Plots of RMSD versus four groups of coupling constants that are sensitive to θ_I: a) Group 1 with $^3J_{N\underline{H},H2}$ and $^3J_{H2,C1'}$; b) Group 2 with $^3J_{N\underline{H},C1}$ and $^3J_{N\underline{H},C3}$; c) Group 3 with $^3J_{C1,C1'}$ and $^3J_{C3,C1'}$; and d) Group 4 with $^3J_{N\underline{H},C1}$, $^3J_{N\underline{H},C3}$, $^3J_{N\underline{H},H2}$, and $^3J_{H2,C1'}$.

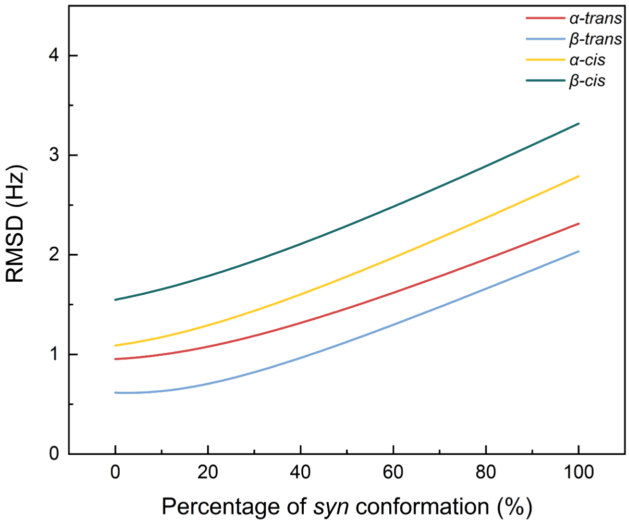

Figure S8. The overall two-state plot of RMSD versus percentage of *syn* conformation using all coupling constants that are sensitive to θ_1.

Supplementary Tables

Table S1. The equilibrium constants $K_{trans/cis}$ of GlcNAc at different solution temperatures and thermodynamic parameters derived from $K_{trans/cis}$.

	$K_{trans/cis}$	
T (°C)	α- pyranoside	β- pyranoside
3.4	171.7	76.5
8.5	139.0	69.6
13.7	130.3	63.9
19.0	124.0	50.1
24.4	109.0	49.3
28.6	101.4	42.6
34.1	89.4	40.8
40.0	88.7	34.8
	Thermodynamic parameter (*cis* → *trans*)	
	α- pyranoside	β - pyranoside
$\Delta H°$ (kJ* mol^{-1})	-12.8 ± 0.92	-15.6 ± 0.94
$\Delta S°$ (J*K^{-1}*mol^{-1})	-3.9 ± 3.13	-20.2 ± 3.21
$\Delta G°$ (kJ*mol^{-1})	-11.6 ± 0.01	-8.9 ± 0.01

Table S2. A summary of NMR experiments that were used to measure ^3J coupling constants.

	**E.COSY**				***J*-Quantitative**	
Multiplet pattern	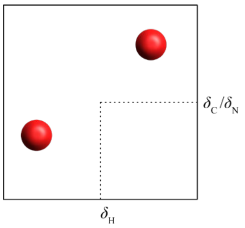				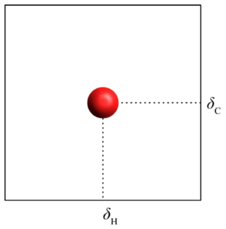	
Coupling constant	$^3J_{N\underline{H},H2}$	$^3J_{H2,C1'}$	$^3J_{N\underline{H},C1}$ and $^3J_{N\underline{H},C3}$	$^3J_{N\underline{H},C2'}$	$^3J_{C1,C1'}$ and $^3J_{C3,C1'}$	$^3J_{C2,C2'}$
Original pulse program	HNCA[HA]-E.COSY	(H)NCAHA[CO]-E.COSY	HNCA[CB]-E.COSY	HNCO[CA]-E.COSY	Spin-echo difference CT-HSQC	*J*-quantitative long-range (H)C(C)H
Adjusted name for aminosugars	HNC2[H2]-E.COSY	(H)NC2H2[C1′]-E.COSY	HNC2[C1/C3]-E.COSY	HNC1′[C2]-E.COSY	/	/

Table S3. Karplus equation coefficients on the formula ^{3}J = **A** + **B** cos θ + **C** cos(2θ) + **D** sin θ + **E** sin(2θ) for θ_1 and the formula ^{3}J = **A** + **B** cos θ + **C** cos(2θ) + **D** cos(3θ) + **E** sin(θ) + **F** sin(2θ) + **G** sin(3θ) for θ_2, as well as torsion angle differences of θ_1 and θ_2.[a]

		θ_1					
		H2-C2-N-H	H2-C2-N-C1'	C1-C2-N-H	C3-C2-N-H	C1-C2-N-C1'	C3-C2-N-C1'
θ_2-*trans*	A	5.08	3.11	2.94	2.57	1.27	1.11
	B	-0.83	-2.02	-1.00	-0.61	-1.31	-0.87
	C	5.02	2.88	3.48	2.92	1.36	1.15
	D	0.03	-0.04	0.12	-0.09	0.12	-0.09
	E	0.06	0.05	-0.27	0.27	-0.07	0.08
θ_2-*cis*	A	5.99	3.19	3.62	3.14	1.37	
	B	-0.59	-2.12	-1.32	-0.78	-1.52	
	C	6.11	3.30	4.13	3.47	1.62	
	D	0.10	-0.02	0.20	-0.16	0.10	
	E	0.12	0.06	-0.46	0.37	-0.15	
Angles	θ_1-*syn* (°)	0	180	120	-120	-60	60
	θ_1-*anti* (°)	180	0	-60	60	120	-120
	Difference (°)[b]	0	180	120	-120	-60	60

		θ_2	
		H-N-C1'-C2'	C2-N-C1'-C2'
θ_1-*anti*	A	1.62	0.39
	B	-2.59	-1.03
	C	1.29	0.36
	D	0.67	0.25
	E	0.02	-0.01
	F	-0.02	0.02
	G	0.003	-0.004
θ_1-*syn*	A	1.62	0.84
	B	-2.59	-2.03
	C	1.29	0.60
	D	0.67	0.13
	E	0.02	0.04
	F	-0.02	-0.02
	G	0.003	0.002
Angles	θ_2-*cis* (°)	180	0
	θ_2-*trans* (°)	0	180
	Difference (°)[c]	180	0

[a] The Karplus equations are from Hu et al.[4]
[b] Compared to H2-C2-N-H.
[c] Compared to C2-N-C1'-C2'.

Table S4. Parameter settings for all NMR experiments.

Sample	Fig.	^{n}J	Experiment	Pulse sequence	NS	Dimensions	SW (ppm)	TD	AQ (ms)	T (°C)	Comments[d]	Exp. time
GlcNAc (unlabeled)	1		1D ^{1}H	zgesgp	8		12	32k	2,272	25[a]		1.5 min
GlcNAc (unlabeled)	S1		1D sel. TOCSY	[b]	256		10	32k	2,726	25	τ_{mix} = 120 ms	17 min
GlcNAc (unlabeled)			2D sel. TOCSY	[b]	64	F2-F1	8×8	2k×128	213×13	25	τ_{mix} = 120 ms	4 h
GlcNAc (unlabeled)	2		1D sel. EXSY	[c]	2k		8	32k	3,412	25	τ_{mix} = 400 ms	2.8 h
UL-^{13}C,^{15}N-GlcNAc	1		^{1}H,^{15}N-HSQC	fhsqcf3gpph	16	F2-F1	17×6	2k×128	100×175	25		48 min
UL-^{13}C,^{15}N-GlcNAc			^{1}H,^{13}C-CT-HSQC	hsqcctetgpsisp	32	F2-F1	8×86	1k×256	107×10	25	2T = 22 ms	3.7 h
UL-^{13}C,^{15}N-GlcNAc			2D (H)C(C)H-TOCSY	hcchdigp3d2	64	F3-F1	8×100	1k×512	107×17	25	τ_{mix} = 11 ms	15.4 h
UL-^{13}C,^{15}N-GlcNAc			2D ^{1}H,^{15}N-HSQC-TOCSY	hsqcdietf3gpsi	16	F2-F1	8×4	1k×128	107×263	25	τ_{mix} = 100 ms	1 h
UL-^{13}C,^{15}N-GlcNAc			2D HNCACB	hncacbgpwg3d	32	F3-F1	7.5×90	1k×128	114×5	25		2 h
UL-^{13}C,^{15}N-GlcNAc	4	$^{3}J_{N\underline{H},H2}$	2D HNCA[HA]-E.COSY	hncaecosgp3d2	32	F3-F1	7.4×14	1k×128	116×30	25		1.5 h
UL-^{13}C,^{15}N-GlcNAc	S4	$^{3}J_{N\underline{H},C1}$	2D HNCA[CB]-E.COSY	hncajcgp3d	64	F3-F1	8×6	8k×128	854×71	25		7 h
UL-^{13}C,^{15}N-GlcNAc	S4	$^{3}J_{N\underline{H},C3}$	2D HNCA[CB]-E.COSY	hncajcgp3d	64	F3-F1	8×6	8k×128	854×71	25		7 h
UL-^{13}C,^{15}N-GlcNAc	S4	$^{3}J_{H2,C1'}$	2D (H)NCAHA(CO)-E.COSY	hncahagp3d	256	F3-F1	7×4	4k×128	487×263	25		25 h
UL-^{13}C,^{15}N-GlcNAc		$^{3}J_{C1,C1'}$, $^{3}J_{C3,C1'}$	2D spin-echo diff. CT-HSQC	hsqcctetgpjclr	64	F2-F1	8×60	2k×256	214×14	25		10.5 h
UL-^{13}C,^{15}N-GlcNAc	4	$^{3}J_{N\underline{H},C2'}$	2D HNCO[CA]-E.COSY	hncoecosgp3d	32	F3-F1	8×2	4k×64	427×106	25		1.5h
UL-^{13}C,^{15}N-GlcNAc	S5	$^{3}J_{C2,C2'}$	2D *J*-quant. long-range (H)C(C)H	hcchetgplr	32	F2-F1	7.5×164	1k×256	114×5	25		4h

[a] A series of experiments were also performed at different temperatures (5-40 °C) to obtain temperature coefficients.
[b] In-house pulse sequences from Alexandersson et al.[5] Bandselective ^{1}H pulses covering *cis* amide protons (6.9 ppm) were achieved with IBURP-2 shapes of 6.45 ms duration and a bandwidth of 770 Hz.
[c] In-house pulse sequence based on the Bruker pulse sequence *selnogp*, with the inclusion of excitation sculpting for water suppression.
[d] The relaxation delay was set to 1.5 s or larger.

References

[1] R. Weisemann, H. Rüterjans, H. Schwalbe, J. Schleucher, W. Bermel, C. Griesinger, *J. Biomol. NMR* **1994**, *4*, 231–240.

[2] A. C. Wang, A. Bax, *J. Am. Chem. Soc.* **1996**, *118*, 2483–2494.

[3] F. Löhr, H. Rüterjans, *J. Biomol. NMR* **1995**, *5*, 25–36.

[4] X. Hu, I. Carmichael, A. S. Serianni, *J. Org. Chem.* **2010**, *75*, 4899–4910.

[5] E. Alexandersson, C. Sandström, L. C. E. Lundqvist, G. Nestor, *RSC Adv.* **2020**, *10*, 32511–32515.
